# Identification of biomarkers for chronic lymphocytic leukemia risk: a proteome-wide Mendelian randomization study

**DOI:** 10.1007/s12672-024-01699-2

**Published:** 2025-01-03

**Authors:** Changyu Jin, Zehong Lu, Yuzhan Chen, Huijie Hu, Miao Zhou, Yanli Zhang, Guifang Ouyang, Tongyu Li, Lixia Sheng

**Affiliations:** Department of Hematology, The First Affiliated Hospital of Ningbo University, No.59 Liu-Ting Road, Ningbo, 315000 People’s Republic of China

**Keywords:** Plasma proteins, Chronic lymphocytic leukemia, Colocalization, Mendelian randomization, FinnGen

## Abstract

**Background:**

Chronic lymphocytic leukemia (CLL) is a common hematologic malignancy. Although previous research has explored associations between plasma proteins and CLL, the causal relationships remain unclear. This study used Mendelian randomization (MR) to investigate the causal relationship between 7156 plasma proteins and CLL risk.

**Methods:**

A two-sample MR analysis assessed the impact of specific plasma proteins on CLL risk, using data from the Finngen Proteomics project (analyzing 828 participants) and the UK Biobank. Additional analyses included colocalization, phenomenon-wide MR, and protein–protein interaction networks.

**Results:**

The study identified nine plasma proteins significantly associated with CLL risk. Increased levels of Peptidyl-prolyl cis-trans isomerase E (PPIE) (OR = 1.66, 95% CI 1.22–2.27, P = 0.001) were associated with an increased risk of developing CLL, whereas Protein O-Mannosyltransferase 2 (POMGNT2) (OR = 0.62, 95% CI 0.41–0.91, P = 0.017) and C–C Motif Chemokine Ligand 14(CCL14) (OR = 0.80, 95% CI 0.67–0.94, P = 0.010) were associated with a reduced risk of CLL. Colocalization analysis suggested that PPIE may share pathogenic variants with CLL (PP.H4 = 0.758). Phenomenon-wide MR analysis of PPIE also indicated associations with other clinical features, including rheumatic diseases and type 2 diabetes. Protein–protein interaction and drug-gene interaction analyses highlighted CDC5L and SNW1 as potential therapeutic targets.

**Conclusion:**

This study identifies nine plasma proteins linked to CLL risk, with PPIE offering new insights into the disease's pathogenesis. Further research is needed to validate these findings and explore their potential as therapeutic targets.

**Supplementary Information:**

The online version contains supplementary material available at 10.1007/s12672-024-01699-2.

## Introduction

Chronic lymphocytic leukemia is a neoplastic disease characterized by the proliferation of small lymphocytes with the markers CD5, CD19, CD20, CD23, as well as the expression of kappa and lambda light chains either on the cell surface or intracellularly [1, 2]. The clinical course of CLL is very variable; While some patients experience stable disease with a favorable prognosis, others may develop refractory and relapsed CLL, leading to reduced survival [3]. Key prognostic factors include IGHV mutations, 17p deletions (del[17p]), and abnormalities in TP53 [4]. From 1990 to 2017, the global incidence of CLL has more than doubled, with over 85% of countries or regions experiencing an upward trend in incidence [5]. Therefore, studying the risk factors associated with CLL represents an extremely important scientific task.

Plasma proteins play a central role in various biological processes, serve as important biomarkers for the diagnosis of specific diseases, and represent important sources of potential therapeutic targets [6]. These proteins can enter the bloodstream through active secretion or cell leakage, providing insights into the current state of human health [7]. Previous studies have confirmed the possible causal relationships between 65 proteins and 52 disease-related phenotypes, suggesting that the occurrence of malignant melanoma is related to proteins such as ASIP and FUT3, while SHISA3 is identified as a risk factor for breast cancer [8].

In the context of CLL, previous research has shown that elevated levels of circulating interleukin-8, interleukin-6, and interleukin-10 correlate with survival outcomes and deleterious disease features [9–11]. Research has also shown that defects in the apoptotic pathway lead to the presence of numerous survival signals in the microenvironment of CLL cells, such as: chemokines and cytokines. These signals enhance the overexpression of nuclear factor-κB and phosphorylated 3-phosphoinositide-dependent protein kinases, particularly members of the Bcl-2 family, and ultimately contribute to tumorigenesis. [12]. Nevertheless, these studies are often limited by factors such as observational designs, small sample sizes, and a limited range of proteins analyzed.

MR (Mendelian randomization) is a well-established method in genetic epidemiology and acts as an instrumental variable in nonexperimental designs to infer causal relationships between modifiable exposures and disease outcomes. This approach exploits the presumed random assignment of genetic variations that are unaffected by disease processes, thereby mitigating confounding factors and reversing causal biases [13]. A recently published genome-wide association study of plasma proteins revealed independent single nucleotide polymorphisms linked to 7156 proteins [14]. Consequently, we integrated two-sample MR analysis, colocalization analysis, phenom-wide MR, and protein–protein interaction analysis to identify potential pathogenic plasma proteins in CLL.

## Methods

### Study design

We implemented a comprehensive analytical framework: 1. We identified disease-related protein targets using a proteomics-wide association study (PWAS) using the two-sample MR method and selected from a database of 7156 proteins from FinnGen. Somascan high-throughput protein analysis technology. 2. We validated the shared coding loci between the identified proteins and CLL using colocalization analysis. 3. We performed Phe-MR analysis of key CLL-related proteins to investigate causal relationships between different disease phenotypes and genetic variants. To reduce data redundancy, we used the UK Biobank SAIGE cohort and excluded diseases with fewer than 500 cases. This resulted in 783 phenotypes being available for Phe-MR analysis. 4. We investigated the potential biological mechanisms of the derived protein targets using PPI network analysis. 5. We identified potential drug targets using a drug-gene interaction database.

### Sources of exposure and outcome data

**Fig. 1:**
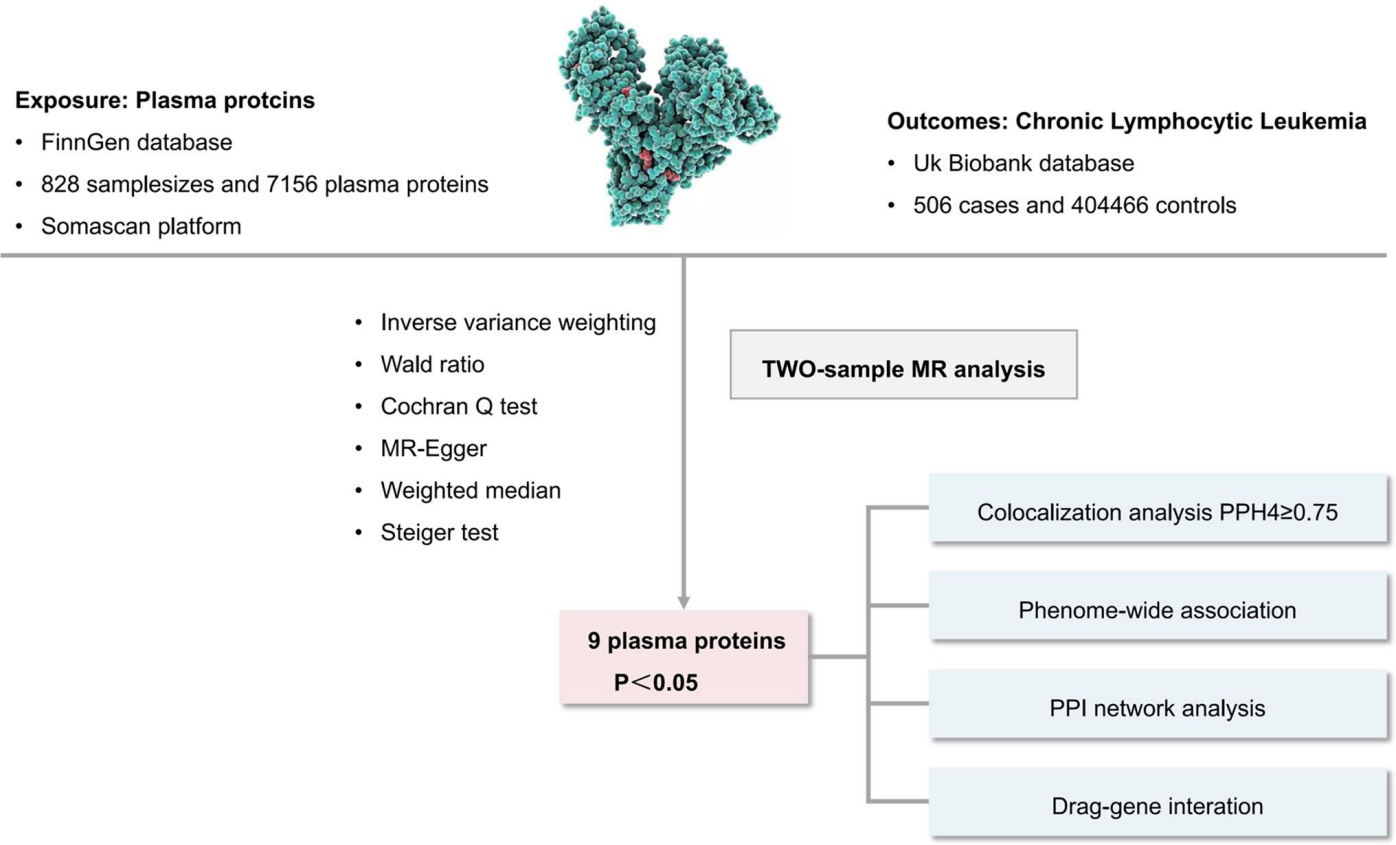
Study design overview

The FinnGen study is a large-scale genomics initiative that analyzes over 500,000 Finnish biobank samples and correlates genetic variations with health data to understand disease mechanisms and susceptibility. This project represents a collaboration between Finnish research institutions, biobanks and international industrial partners [14]. FinnGen used Somascan's high-throughput protein analysis technology to perform proteomic analyzes on 828 plasma samples, collecting data on 7156 proteins (https://r10.finngen.fi/). Result data comes from the UK Biobank database, the world's largest biobank, containing phenotypic and genomic data from over 500,000 individuals. These data facilitate research into gene-phenotype or gene-disease relationship s and help researchers identify drug targets or biomarkers for disease diagnosis and treatment. Disease GWAS from this database used scalable and accurately implemented generalized linear mixed models (SAIGE V.0.29) to address imbalanced case–control ratios [15] (https://pheweb.org/UKB-SAIGE/). Public data on CLL includes 506 cases and 404,466 controls **(**Fig. 1).

### Mendelian randomization analysis

MR analysis is based on three basic assumptions: (1) genetic variation is strongly associated with plasma proteins; (2) Genetic variation is not influenced by confounding factors that could influence the relationship between plasma proteins and CLL. and (3) genetic variation influences CLL solely through its influence on plasma proteins, without other pathways. We selected pQTLs as instrumental variables (IVs) based on the following criteria: (1) SNPs within ± 10,000 kb of gene regions; (2) Selection of highly associated SNPs (P < 5 × 10^–8^) for 7156 plasma proteins across the genome; (3) an LD threshold (r^2^) of 0.001 and a distance of 10,000 kb to ensure independent SNPs and minimize LD effects; 4) R^2^ and F statistics $\left( {{\text{R}}^{{2}} \,{ = }\,{2} \times {\text{MAF}} \times \left( {{1} - {\text{MAF}}} \right) \times \beta^{{2}} \,{\text{;F}}\,{ = }\,{\text{R}}^{{2}} \times \frac{{\left( {{\text{N}} - {2}} \right)}}{{\left( {{1} - {\text{R}}^{{2}} } \right)}}} \right)$ for estimating the strength of genetic tools, where R^2^ represents the Proportion of variation in protein levels explained by each genetic tool [16]. We selected the protein with the highest R^2^ value for repeated proteins in the study. For SNP harmonization, the “Harmonize Data” function in the TwoSample MR package was used.

Subsequently, methods such as Inverse variance weighted(IVW), Wald ratio, MR Egger and weighted median (WM) were used to evaluate causal relationships between plasma proteins and CLL [17]. Variables were further classified as cis-pQTLs or trans-pQTLs based on the following criteria: pQTLs that were within 1 Mb of the transcription start site of a protein-coding gene were defined as cis-pQTLs, while those outside this region were referred to as trans-pQTLs. The Wald ratio and delta method were used to estimate odds ratios (ORs) and corresponding confidence intervals (CIs) for proteins with only one cis-pQTL.Estimates were obtained using IVW method for proteins with multiple cis-pQTLs. The Wald ratio method is suitable as a basic MR approach for cases involving one or more genetic instruments. It estimates the causal effect of exposure on the outcome by calculating the ratio of correlation coefficients between each SNP and the exposure and the coefficients between each SNP and the outcome. The IVW method provides the highest statistical power and is often used as the primary analysis method in MR studies with multiple genetic variations, assuming that all genetic variables are valid instruments. The weighted median method assumes that at least 50% of instruments are valid, whereas MR Egger serves as a complementary method in MR studies to test and adjust for pleiotropy MR Egger is sensitive to outliers and provides causal estimates even when all IVs are invalid [18]. MR-Egger regression was used to detect horizontal pleiotropy when the number of SNPs was three or more, with P values above 0.05 indicating no pleiotropy, while heterogeneity was checked using the Cochran Q test, with P values above 0.05 indicating no pleiotropy-influencing heterogeneity [19]. To avoid reverse causality, the MR-Steiger test was used [20]. However, due to the limited number of instrumental variable SNPs, the MR-PRESSO method could not be used to detect and correct horizontal pleiotropy. In this study, all analyses were performed using the “TwoSampleMR” and “coloc”packages in R version 4.2.2. To control false positives when testing multiple hypotheses, false discovery rate (FDR) was applied to eliminate statistical bias from multiple comparisons [21].

### Colocalization analysis

Colocalization analysis is used to test whether the association of the identified protein with CLL shares the same causal variants. This analysis is based on a Bayesian model with posterior probabilities for five hypotheses (PPH): (1) no association with any feature (H0), (2) association only with feature 1 (H1), (3) association only with Trait 2 (H2), (4) different causal variants associated with both traits (H3), and (5) the same causal variant associated with both traits (H4). The “coloc.abf” algorithm uses standard parameters (a priori probabilities of SNP association: with feature 1, p1 = 1 × 10^–4^ with feature 2, p2 = 1 × 10^–4^; with both features, p12 = 1 × 10^–5^) [22]. An association between the identified protein and CLL is defined as colocalized if PPH4 > 0.75, while PPH4 > 0.5 indicates moderate colocalization [16].

### Phenome-wide MR analysis

To further evaluate potential drug targets and predict adverse drug events, we performed Phe-MR analysis of key proteins associated with CLL [23]. To avoid data redundancy, we used the UK Biobank cohort, which includes a sample size of up to 408,961 individuals. We excluded disease phenotypes with fewer than 500 cases. The researchers used SAIGE v.0.29 software to conduct 30 GWAS analyses, using a generalized mixed model approach to eliminate potential confounds caused by unbalanced case–control distributions. Phenotypic outcomes for diseases or conditions were defined using the “PheCodes” system, which organizes codes from the International Classification of Diseases and Related Health Problems (ICD-9/−10) that correspond to specific phenotypic expressions [15].

### PPI network analysis

To validate the interactions of the identified proteins, we performed protein–protein interaction (PPI) network analysis on proteins significantly associated with CLL (q < 0.05). All PPI analyzes were performed using the STRING database version 11.5 (https://string-db.org/) with a minimum interaction score threshold of 0.4.

### Drug-gene interaction

To determine whether the identified proteins could serve as potential therapeutic targets, we utilized the Drug-Gene Interaction Database (https://www.dgidb.org/), which contains information on drug-gene interactions and druggable genes from publications and databases and online resources.

## Results

### Potential positive proteins influencing CLL

**Fig. 2:**
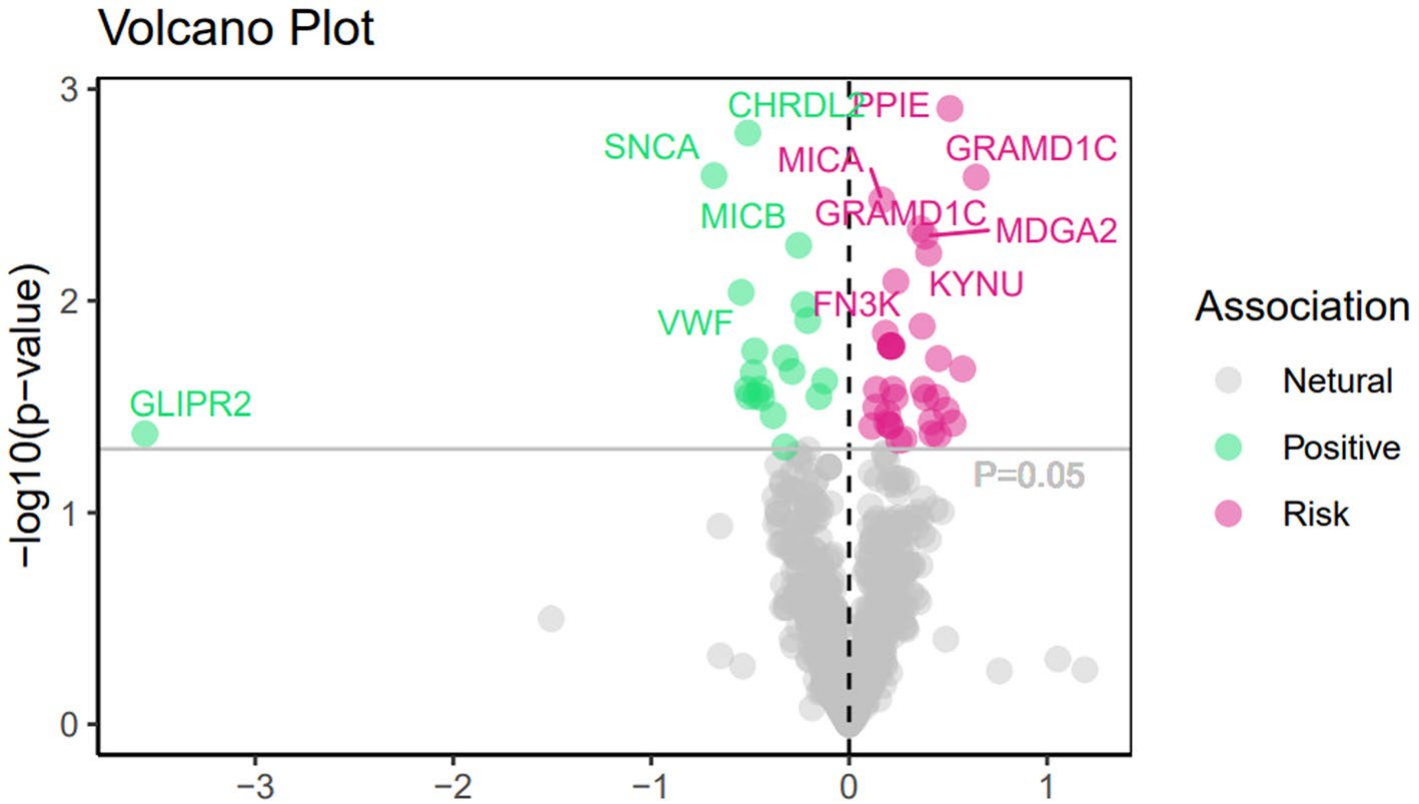
MR correlation analysis of plasma proteins with chronic lymphoid leukemia, Pval < 0.05 was considered as significant association

**Fig. 3:**
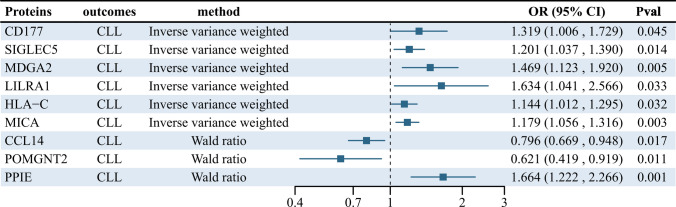
Association of protein expression in the blood with CLL risk. The forest map for estimates of the relationship between genetically predicted protein levels and CLL

A comprehensive analysis of the causal relationship between 7,156 plasma proteins and CLL was performed using the IVW or Wald ratio method. The F value, which indicates the strength of the association between genetic variants and exposure, ranged from 30 to 1,380 and showed no risk of weak instrument bias. Although all plasma proteins showed no statistically significant effect on CLL after FDR correction (p-fdr < 0.05), there are still nine unmatched plasma protein phenotypes with low p-value that are noteworthy (Fig. 2).Specifically, type III receptor tyrosine kinase (CD117) (OR = 1.32, 95% CI 1.00–1.72, P = 0.045), MHC class I polypeptide-related sequence A (MICA) (OR = 1.18, 95% CI 1.06–1.32, P = 0.003), Sialic Acid Binding IG-Like Lectin 5 Recombinant Protein (SIGLEC5) (OR = 1.20, 95% CI 1.03–1.39, P = 0.014), MAM Domain Containing Glycosylphosphatidylinositol Anchor 2 (MDGA2) (OR = 1.47, 95% CI 1.12–1.92, P = 0.005), Leukocyte Immunoglobulin-Like Receptor A1 (LILRA1) (OR = 1.63, 95% CI 1.04–2.57, P = 0.033), Human Leukocyte Antigen C (HLA-C) (OR = 1.14, 95% CI 1.01–1.29, P = 0.032) and Peptidyl-prolyl cis–trans isomerase E (PPIE) (OR = 1.66, 95% CI 1.22- 2.27, P = 0.001) were associated with an increased risk of CLL. In contrast, protein O-linked mannose Protein O-Mannosyltransferase 2 (POMGNT2) (OR = 0.62, 95% CI 0.41–0.91, P = 0.017) and C–C motif chemokine 14 (CCL14) (OR = 0.80, 95% CI 0.67–0.94, P = 0.011) were associated with a reduced risk of CLL (Fig. 3). More details for the association of proteins with CLL are presented in Supplementary Tables 1–3.

### Colocalization results

**Fig. 4:**
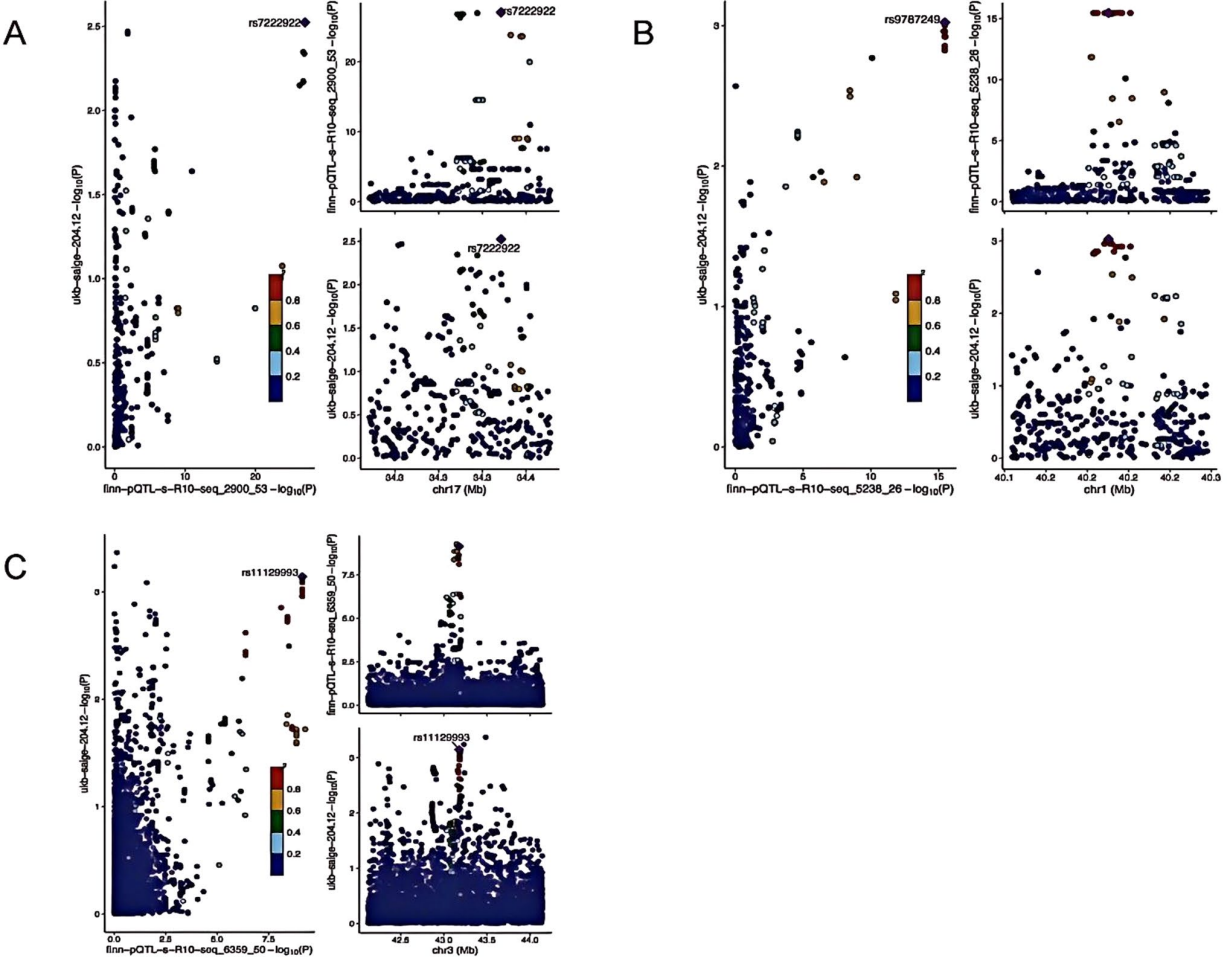
Genetic colocalization of CLL (**A**) CCL14 (**B**) PPIE (**C**) POGNT2. In this view, each dot is a genetic variant. Te SNP with the most notable P value with CLL is marked, and the colors of other SNPs depends on the digit size ordering of linkage disequilibrium (r2). SNPs with missing linkage disequilibrium information are also coded dark blue. In the LocusZoom plots, -log10 (P.gwas) for links with CLL risk are on the x-axes, and -log10 (P.pqtl) for relationship with the protein levels on the y-axes

To validate genetic colocalization, PP was assessed. A PP.H4 ≥ 0.75 was defined as positive colocalization, while a PP.H4 ≥ 0.50 was defined as moderate colocalization (Supplementary Tables 4). In this study, gene colocalization analysis of five proteins suggested that PPIE may have a pathogenic variant in this region (PP.H4 = 0.758); POMGNT2 (PP.H4 = 0.603) and CCL14 (PP.H4 = 0.549) showed moderate colocalization with CLL (Fig. 4).

### Phenome-wide MR analysis results

**Fig. 5:**
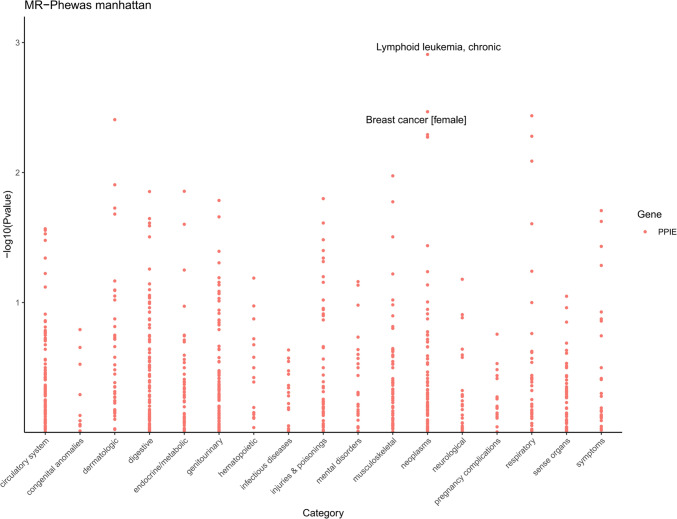
The phenome-wide MR analysis for PPIE

To comprehensively elucidate the influence of the CLL-associated PPIE protein on other clinical features, we performed PHEWAS on the UK Biobank dataset, which included 1403 diseases. Our study shows that PPIE is associated with 45 traits (Pval < 0.05). In particular, PPIE shows a positive correlation with 22 clinical characteristics, suggesting that PPIE not only increases the risk of CLL, but also the associated risks of endocrine disorders such as rheumatoid diseases (OR = 1.14, 95% CI 1.01–1.29. Pval = 0.014) and type 2 diabetes (OR = 1.30, 95% CI 1.05–1.62, Pval = 0.040). Furthermore, PPIE is negatively correlated with 23 clinical features, suggesting a potential protective effect (Fig. 5). Consequently, any development of the plasma protein PPIE as a therapeutic target for CLL must take into account its potential side effects and safety.

### PPI networks and drug-gene interactions.

**Fig. 6:**
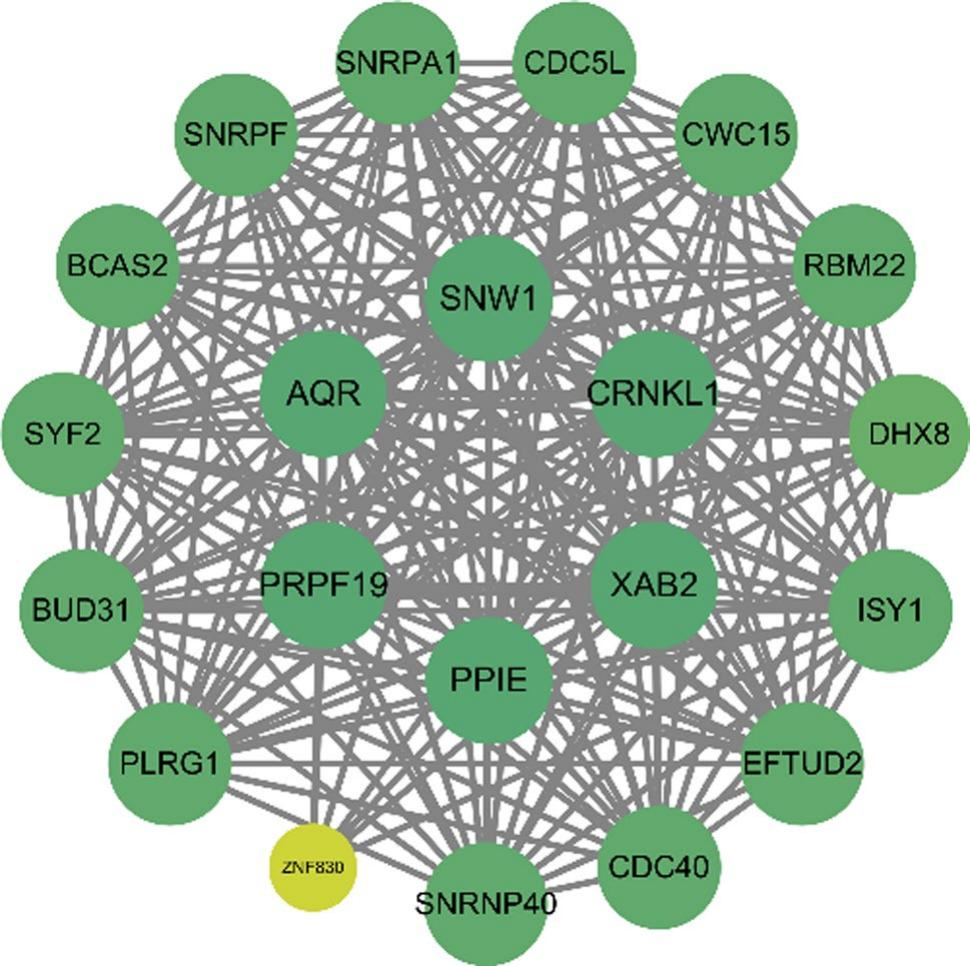
Protein–protein interaction (PPI) network among identified protein targets and suggestive protein targets

Using the STRING interaction gene and protein search tool, we analyzed the protein–protein interaction network (PPI) of 885 DEGs. The CytoHubba plugin grading algorithm identified 20 protein genes that interact with PPIE: AQR, ZNF830, ISY1, SYF2, CWC15, SNRPF, SNRNP40, DHX8, SNW1, BUD31, PLRG1, PPIE, XAB2, RBM22, CDC5L, BCAS2, PRPF19, CDC40, EFTUD2 and CRNKL1 (Fig. 6). We then used the drug-gene interaction database to identify two potential therapeutic targets for CLL–CDC5L and SNW1–from the 21 proteins, including PPIE, within the PPI network.

## Discussion

By integrating plasma proteomics with genome-wide association data, this study systematically identified plasma proteins associated with chronic lymphocytic leukemia through MR and Bayesian colocalization analyzes with the aim of uncovering potential drug targets. The discovery and evaluation of diverse therapeutic targets provides additional options for CLL treatment, including small molecule inhibitors, monoclonal antibodies, and other biologics. Nevertheless, individual patient responses may vary, highlighting the need to explore new strategies. Our MR analysis identified nine CLL-associated proteins, seven (CD177, SIGLEC5, MDGA2, LILRA1, HLA-C, MICA, PPIE) significantly increased CLL risk, while POGNT2 and CCL14 were associated with reduced risk. Colocalization analysis suggests that PPIE may be a promising therapeutic target. Further investigations using Phe-MR are planned to evaluate possible side effects and safety of targeted PPIE.

PPIE, an enzyme from the peptidyl prolyl isomerase (PPIase) family, was recognized as a CLL-related plasma protein in this study. The PPIase family plays a crucial role in various diseases. For example, PPIA, a prominent member called cyclophilin A (CyPA), facilitates the isomerization of proline residues in proteins. Previous studies using single-cell transcriptomics revealed high PPIA expression in primary refractory multiple myeloma patients and those unresponsive to KYDAR chemotherapy. CRISPR knockout of PPIA in the RPMI-8226 cell line showed that PPIA depletion or treatment with the CyPA inhibitor CsA increased sensitivity to the proteasome inhibitor CFZ, suggesting the role of PPIA in drug resistance and the potential for overcoming them [21]. B-cell malignancies such as multiple myeloma (MM) and CLL often colonize the bone marrow, where the microenvironment plays a critical role in their proliferation and protection [24–26]. Exogenous CyPA (eCyPA) secreted by bone marrow endothelial cells promotes proliferation and homing of MM cells by binding to the receptor CD147 [27].In addition to MM cells, eCyPA also promotes the migration of chronic lymphocytic leukemia (CLL) and lymphoplasmacytic lymphoma (LPL) cells, both of which express CD147 and tend to colonize the bone marrow.This suggests that PPIE, a member of the same family as PPIA, may play an important role in cellular signaling pathways. PPIE encodes an N-terminal RNA recognition motif (RRM) and a C-terminal isomerase domain [28]. It regulates chromatin modification, transcription and pre-mRNA splicing by directly interacting with the PHD3 domain of the histone reader MLL1 and associating with the XAB2 complex [29–33]. PPIE interacts with various splicing factors, including SF3B4, SF3A2, snRNPA’, B/B’ and SmD3, and may influence splicing patterns and thus protein production in B cells [34–36].

CLL is characterized by the pathological accumulation of mature B cells. Cellular stresses such as oxidative stress, DNA damage, and protein misfolding may contribute to disease progression by affecting apoptosis, cell cycle regulation, DNA repair mechanisms, and immune evasion. PPIE, as a molecular chaperone, is central to protein folding and catalyzes the isomerization of proline residues to ensure correct protein folding under stressful conditions, thereby preventing misfolding and protecting cells from damage [37]. The PPIase family also regulates the cell cycle and influences proliferation and aging, with CyPA being involved in the aging of hematopoietic stem cells. These potential functions and roles suggest that abnormal expression of PPIE plays an important role in the pathogenesis of CLL. However, further experimental validation is required to evaluate the potential of PPIE as a therapeutic target [38].

MICA, also known as MHC Class I Polypeptide-Related Sequence A, is a stress protein that is rarely expressed in normal cells, except in the gastrointestinal epithelium, endothelial cells and fibroblasts. High MICA expression is detected on various tumor cells, including non-small cell lung cancer, colon cancer, breast cancer and leukemia, which is consistent with our MR analysis. Tumor cells can evade immune surveillance by degrading MICA, leading to its internalization by NK cells via NKG2D, thereby silencing NK cell responses [39]. Therefore, anti-MICA antibodies represent a potential therapeutic target for CLL. CD177, also known as NB1 or HNA-2 antigen, is a GPI-anchored surface protein typically expressed on neutrophils. CD177 mediates neutrophil adhesion and migration through interactions with PECAM-1 and β2 integrin. It is considered a valuable marker in myeloproliferative diseases [40]. Although the association between CD177 and CLL risk has not been extensively studied, our findings suggest a potentially positive correlation. Studies have shown that CD177-deficient Treg cells reduce tumor growth and TI-Treg frequency in mice [40]. Although the strict correction thresholds are not met, several proteins with low p values ​​are notable in our study. The SIGLEC protein family has been shown to contribute to the establishment of an immunosuppressive microenvironment in tumor cells by inducing a pro-cancer phenotype in tumor-associated macrophages and inhibiting immune cell activation [41]. Similar to CD177, there is limited research on the association of POGNT2 and CCL14 with CLL risk. Our MR results indicate a negative correlation between POGNT2, CCL14 and CLL risk, which warrants further investigation to understand the role of these three proteins in the development of CLL.

However, this study has limitations..Gene expression is highly complex and is influenced by various factors, including environmental conditions. The proteomic analysis in this study is limited to pQTL data from individuals of European ancestry, potentially introducing a bias for non-European populations. Furthermore, our main data source, plasma proteomics from the FinnGen cohort, is based on hypothesis-based genomic methods and does not include other relevant tissue systems, leading to selection bias. Unique pQTLs may have been missed, and unknown rare variants and complex counteractions may play an important role in genetics, warranting investigation in larger future studies.Regarding the assessment of druggability, it should be emphasized that this study identifies potential drug targets primarily through Mendelian randomization and colocalization evidence. There are currently no reports of drugs targeting this candidate for the treatment of CLL. Furthermore, the association of PPIE with various other clinical features raises concerns about possible off-target side effects, complicating its suitability as a drug target. Therefore, we conducted a protein interaction network analysis where alternative proteins within common signaling pathways may provide therapeutic options when the identified proteins are considered untreatable. This is because potential targets do not act in isolation but rather interact within a complex network of interconnected signaling pathways that contribute to disease pathology. In addition, some studies have shown that β2-microglobulin correlates with tumor burden, disease stage and prognosis in CLL, abnormalities in the secretion of plasma proteins such as IL-10 and TNF-α may be involved in the immune regulation of the tumor microenvironment and thereby tumor cell proliferation and affect survival. However, in the absence of confirmed tissue-specific roles in CLL, the use of plasma proteins as surrogates limits their explanatory power in directly implicating disease mechanisms. Therefore, although our method is based on careful hypotheses and considerations, it is crucial to integrate evidence from Mendelian randomization and colocalization, protein interactions with recently identified genes, as well as gene functionality and tissue-specific expression patterns to accurately determine disease-associated drug target genes. Furthermore, further clinical and microexperimental studies are essential to refine the evidence and confirm the potential of PPIE as a therapeutic target in CLL.

## Conclusion

This study identified nine plasma proteins linked to CLL risk, with PPIE offering new insights into CLL pathogenesis. These findings establish a solid foundation for further research into the genetic and functional mechanisms underlying CLL and highlight potential biomarkers for developing novel therapeutic strategies. Future research should focus on validating the specific roles of these proteins in CLL onset and progression, and assessing their potential as therapeutic targets, to enhance the development of precise diagnostic and treatment strategies for CLL.

## Supplementary Information

Supplementary Material 1. Table 1. Details for the SNPs related to proteins. Table 2. The association between proteins and CLL in the MR analysis. Table 3. The heterogeneity for the MR analysis based on Cochran Q test. The MR Egger results to assess the horizontal pleiotropy. Table 4. The results of colocalization analysis for proteins. Table 5. The phenome-wide MR analysis for PPIE

## Data Availability

The original contributions presented in the study are included in the article/ Supplementary Materials. Further inquiries can be directed to the corresponding authors.
